# Epiplakin Deficiency Aggravates Murine Caerulein-Induced Acute Pancreatitis and Favors the Formation of Acinar Keratin Granules

**DOI:** 10.1371/journal.pone.0108323

**Published:** 2014-09-18

**Authors:** Karl L. Wögenstein, Sandra Szabo, Mariia Lunova, Gerhard Wiche, Johannes Haybaeck, Pavel Strnad, Peter Boor, Martin Wagner, Peter Fuchs

**Affiliations:** 1 Department of Biochemistry and Cell Biology, Max F. Perutz Laboratories, University of Vienna, Vienna, Austria; 2 Department of Internal Medicine III and IZKF, University Hospital Aachen, Aachen, Germany; 3 Institute of Pathology, Medical University of Graz, Graz, Austria; 4 Division of Nephrology and Institute of Pathology, RWTH University of Aachen, Aachen, Germany; 5 Department of Internal Medicine I, University Medical Center Ulm, Ulm, Germany; University of Szeged, Hungary

## Abstract

Epiplakin, a member of the plakin protein family, is exclusively expressed in epithelial tissues and was shown to bind to keratins. Epiplakin-deficient (EPPK^−/−^) mice showed no obvious spontaneous phenotype, however, EPPK^−/−^ keratinocytes displayed faster keratin network breakdown in response to stress. The role of epiplakin in pancreas, a tissue with abundant keratin expression, was not yet known. We analyzed epiplakin’s expression in healthy and inflamed pancreatic tissue and compared wild-type and EPPK^−/−^ mice during caerulein-induced acute pancreatitis. We found that epiplakin was expressed primarily in ductal cells of the pancreas and colocalized with apicolateral keratin bundles in murine pancreatic acinar cells. Epiplakin’s diffuse subcellular localization in keratin filament-free acini of K8-deficient mice indicated that its filament-associated localization in acinar cells completely depends on its binding partner keratin. During acute pancreatitis, epiplakin was upregulated in acinar cells and its redistribution closely paralleled keratin reorganization. EPPK^−/−^ mice suffered from aggravated pancreatitis but showed no obvious regeneration phenotype. At the most severe stage of the disease, EPPK^−/−^ acinar cells displayed more keratin aggregates than those of wild-type mice. Our data propose epiplakin to be a protective protein during acute pancreatitis, and that its loss causes impaired disease-associated keratin reorganization.

## Introduction

Epiplakin, a large 725 kDa protein encoded by a single exon, was originally isolated as an autoantigen from a patient suffering from subepidermal blistering [1], [2]. Subsequent analyses revealed epiplakin to consist entirely of plakin repeat domains (PRDs). Its 16 consecutive PRDs in mouse and 13 in the human protein qualified epiplakin as a member of the plakin protein family [3], [4]. Plakins represent established cytoskeletal organizers that bind to and interconnect cytoskeletal filaments. The lack of other protein domains typically present in plakins makes epiplakin a unique member of this protein family (for review, see [5]). Epiplakin expression is restricted to epithelia including simple epithelial tissues of the digestive system [3], [4], e.g. of liver and pancreata of mice [6], [7]. The only direct binding partners for epiplakin conclusively identified so far are intermediate filament proteins including epithelial keratins [4], [8], [9]. In mice, null mutations of other plakins (e.g. plectin) resulted in severe phenotypes including skin blistering [5]. We and others generated epiplakin knock-out (EPPK^−/−^) mice, which, surprisingly, showed no obvious spontaneous or stress-induced *in vivo* phenotype [10], but accelerated migration of keratinocytes *ex vivo* [11]. Subsequent analyses revealed that epiplakin protects from hyperphosphorylation-induced keratin disruption in keratinocytes, thereby indicating a function in stress response [12]. Epiplakin interacts with keratin 8 and 18 (K8 and K18), the main keratins expressed in simple epithelia [8]. In pancreatic acinar cells, K8 and K18 form the major keratin heteropolymers [13], whereas minor levels of K8/K19 are found close to the apicolateral membrane [14]. Interestingly, mice lacking acinar keratins did not show increased susceptibility to experimentally induced pancreatitis [14]. On the other hand, excessive overexpression of human K8 or K8/K18 in mouse pancreata led to pancreatic disorders resembling chronic pancreatitis [15], [16]. These data showed that significant overexpression of keratins can cause pancreatic injury. Additionally, upregulation of keratin expression is known to be a consequence of experimental pancreatitis, which is accompanied by dramatic reorganization of the keratin filament system [14], [17]. Interestingly, in preliminary experiments we found that in response to caerulein-induced pancreatitis increased epiplakin expression paralleled that of keratin. These findings suggest a potential role of epiplakin in pancreatic injury. To address this question, we analyzed the pancreatic expression pattern of epiplakin in more detail and compared the susceptibility of wild-type and EPPK^−/−^ mice to caerulein-induced acute pancreatitis and their pancreatic acinar keratin filament network organization.

## Materials and Methods

### Animals/Pancreatitis induction

For the animal experiments described in the manuscript an animal experiment approval was granted by the Austrian ministry of science, research and economy. The following approval covers the experiments performed in the manuscript: „Untersuchung der in vivo Funktion von Epiplakin in einfachen Epithelien von Leber, Darm und Bauchspeicheldrüse“; BMWF-66.006/0017-II/3b/2011. No institutional animal care, user committee, or ethics committee was existing at the University of Vienna at that time or was a legally requirement for this approval. The relevant legislation for the approval was: TVG, BGBI. Nr. 501/1989 i.d.F. BGBI. I Nr. 162/2005.

Animals were excluded from the experimental research in case of showing any of the following symptoms: apathy (inactivity, no reaction to external stimuli), no food or water intake, dehydration, breathing difficulties, cyanosis, motor or neurological impairments (signs of paralysis, persistent tremor, cramps), non-physiological abnormal posture (“crouched position”), heavy fluid discharge from orifices, persistent spontaneous stress, or auto mutilation. At the end of the experiment, the animals were anaesthetized with isoflurane and killed by cervical dislocation.

All experiments were performed using 2–4 months old sex-matched littermate mice. The previously described EPPK^−/−^ mice [10] were backcrossed into the C57BL/6J background (N10). Pancreatitis was induced in adult wild-type and EPPK^−/−^ mice. Mice received 7 hourly intraperitoneal injections of 50 µg/kg ceruletide diethylamine (Caslo, Lyngby, Denmark) or PBS as control and were sacrificed at given timepoints after the initial injection.

### Pancreas protein lysates

Mice were anaesthetized with 150 µl isoflurane and killed by cervical dislocation. The pancreas was removed, lysed in 500 µl lysis buffer [20 mM Tris pH 7.5; 150 mM NaCl; 5 mM MgCl_2_; 5 mM EDTA; 1% Triton X-100; 0.5% NP-40; Protease Inhibitor Cocktail (Roche Applied Science, Indianapolis, IN); Phosphatase Inhibitor Cocktails (Sigma, St Louis, MO); 0.5 mg/ml DNAse I; 0.2 mg/ml RNAse A; 1 mM PMSF] and incubated for 10′ at RT. The lysate was mixed with sample buffer (400 mM Tris pH 6.8; 10% SDS; 50% glycerol; 500 mM DTT; 0.1% bromphenolblue), boiled at 95°C for 5′ and the cellular debris was removed by centrifugation.

### Antibodies

The following primary antibodies were used for immunoblotting (IB), immunofluorescence microscopy (IFM) and immunohistochemistry (IHC): affinity-purified rabbit antibodies to epiplakin (IB 1∶10.000, IFM 1∶1.000, IHC 1∶1000) [4]; mouse monoclonal antibody (mAb) to K18 (Ks18.04, Progen; IB 1∶500); rabbit mAb to K7 (R17-S, DB Biotech, Kosice, Slovakia; IFM 1∶100); rat mAb to K8 (Troma I, Developmental Studies Hybridoma Bank, University of Iowa, Iowa City, IA [18]; IB 1∶500, IFM 1∶50, IHC 1∶50); mAb to desmoplakin (DP I/II 236.23.1, Progen; IFM undiluted); mAb to occludin (OC-3F10, Life Technologies, Carlsbad, CA; IFM 1∶200); mAb to e-cadherin (36/E-Cadherin, BD Biosciences, Franklin Lakes, NJ; IFM 1∶35); and rat mAb to K19 (Troma III, Developmental Studies Hybridoma Bank, University of Iowa [18]; IB 1∶500). Secondary antibodies were donkey anti-rat rhodamine red (RRX)-conjugated IgGs (IFM 1∶200), donkey anti-rabbit IgGs conjugated to Alexa Fluor 488 (A-488) and RRX (IFM 1∶500, 1∶200), donkey anti-mouse RRX and A-488-conjugated IgGs (IFM 1∶200, 1∶500), goat anti-mouse/rabbit/rat horseradish peroxidase (HRP)-conjugated IgGs (IB 1∶10.000, 1∶20.000, 1∶20.000) (all from Jackson Immunoresearch, West Grove, PA); rabbit anti-rat (IHC 1∶200) and goat anti-rabbit biotin-conjugated IgGs (IHC 1∶500) (both from Dako, Glostrup, Denmark).

### Coomassie staining and Immunoblotting

Protein lysates were separated by SDS PAGE (6–10%). Gels were either stained with Coomassie brilliant blue or - for IB - proteins were transferred to nitrocellulose membranes. The membranes were blocked with 5% milk powder in PBS with 0.1% Tween-20 and subsequently incubated with primary antibodies followed by incubation with HRP-conjugated secondary antibodies. Detection was carried out using a Fusion FX chemiluminescence system (PEQLAB, Erlangen, Germany). Quantification of protein bands was performed using QuantiScan version 1.5 software (Biosoft, Cambridge, UK).

### Quantitative Real Time RT-PCR

Total RNA was isolated using RNeasy mini kit (Qiagen, Valencia, CA) and transcribed into cDNA with a Superscript II reverse transcriptase (Invitrogen, Carlsbad, CA). Quantitative realtime PCR was performed with a Sequence Detection System (Applied Biosystems 7300 fast Real Time PCR system) using primers specific for murine epiplakin (5′-ATGGGTACCCCTGGTTTTTC-3′ and 5′-CAGGGTGTGGAAAGTGGTCT-3′). Samples were analyzed in duplicates. L7 ribosomal protein was used as internal control (primers: 5′-GAAAGGCAAGGAGGAAGCTCATCT-3′ and 5′-AATCTCAGTGCGGTACATCTGCCT-3′) and cDNA levels were normalized for equal expression of L7 in all tested samples. Epiplakin transcript levels relative to L7 were determined and reported as means ± s.e.m.; n = 3.

### Immunohistochemistry and Immunofluorescence microscopy on paraffin-embedded pancreas sections

The removed pancreata were fixed in 4% buffered formaldehyde (Agar, Stansted, UK) for 24 h, embedded, cut into 4 µm thick sections and stained with hematoxylin and eosin (H&E). For IHC and IFM, sections were subjected to antigen retrieval by boiling in EDTA buffer. Blockings of endogenous peroxidases and unspecific antigen interactions were performed with 3% H_2_O_2_ and 2% BSA, respectively. Afterwards, sections were incubated with primary antibodies for 1 h at room temperature. Fluorophore-conjugated antibodies were used for IFM. Primary antibodies used in IHC were visualized by biotinylated secondary antibodies and diaminobenzidine (DAB) detection using the animal research kit (Dako).

### Patients

Human pancreatic tissues were obtained from the databank of the Institute of Pathology in Aachen and processed in an anonymous manner. These tissues were previously used for diagnostic purposes. Three histomorphologically normal pancreata and three specimens with acute necrotizing pancreatitis were immunohistochemically stained for epiplakin and K8.

### Histopathological score

Pancreatic morphology was scored for edema, necrosis and leukocyte infiltration as described before [19] in a blinded manner.

### Measurements of lipase/amylase levels

Serum lipase/amylase levels were measured at Invitro laboratories (Invitro, Vienna, Austria).

### Electron microscopy

Pancreata of EPPK^−/−^ and littermate control mice were cut into small blocks and fixed in 2% glutaraldehyde/2% formaldehyde in 0.1 mol/L cacodylate buffer and embedded in epoxy resin. The areas for further examination, i.e. the blocks cut from the specimen, were selected randomly. Ultrathin sections for electron microscopy were stained with uranyl acetate and lead citrate. The slides were imaged using the EM10 electron microscope (Zeiss, Carl Zeiss Inc., Jena, Germany), digitalized and evaluated for zymogen granule size using ImageJ software (National Institutes of Health, Bethesda, MD).

### Amylase secretion assay

Total amylase content of acini was determined by homogenizing pancreata in a glass dounce homogenizer in lysis buffer (25 mM Tris, pH 9; 0.01% Triton X-100). Pancreatic lobules were isolated as described in [20] or [21].The spontaneous release and stimulated secretion response were determined by measuring the extent of amylase release from isolated acini after incubation in oxygen-saturated incubation solution (20 mM HEPES, pH 7.4; 120 mM NaCl, 5 mM KCl, 1.2 mM MgCl_2_, 2 mM CaCl_2_, 14 mM glucose, Eagle’s minimal essential medium amino acid supplement and 2 mM L-glutamine) at 37°C for 30, 60, and 90 min in the presence or absence of 0.1 nM ceruletide diethylamine (Caslo).

### Image acquisition

IFM was performed using a confocal laser-scanning microscope (Zeiss LSM 710) equipped with Plan-Apochromat 63x and 40x/1.4 NA oil-immersion objective lenses, using the ZEN software. Deconvolution was performed using Huygens deconvolution software (SVI, Hilversum, Netherlands). H&E as well as IHC images were acquired with an Axiophot microscope (Carl Zeiss Inc.) equipped with either Plan-Neofluar 10x/0.3 NA or Plan-Neofluar 20x/0.5 NA objectives using AxioVision 4.8 software (Carl Zeiss Inc.). Further image adjustments (brightness and contrast) were done with Photoshop CS5 (Adobe Systems, San Jose, CA) or ImageJ software (National Institutes of Health).

### Biostatistical analyses

Values of two groups were compared using unpaired two-tailed Student’s t-test (α = 0.05) or Mann–Whitney–Wilcoxon test (α = 0.05) based on the result of a normality test. All statistical analyses were performed using GraphPad Prism 5 (GraphPad Software, Inc., La Jolla, CA).

## Results

### Epiplakin is expressed in ductal and acinar cells of the exocrine pancreas and its subcellular localization depends on keratin filaments

**Figure 1:**
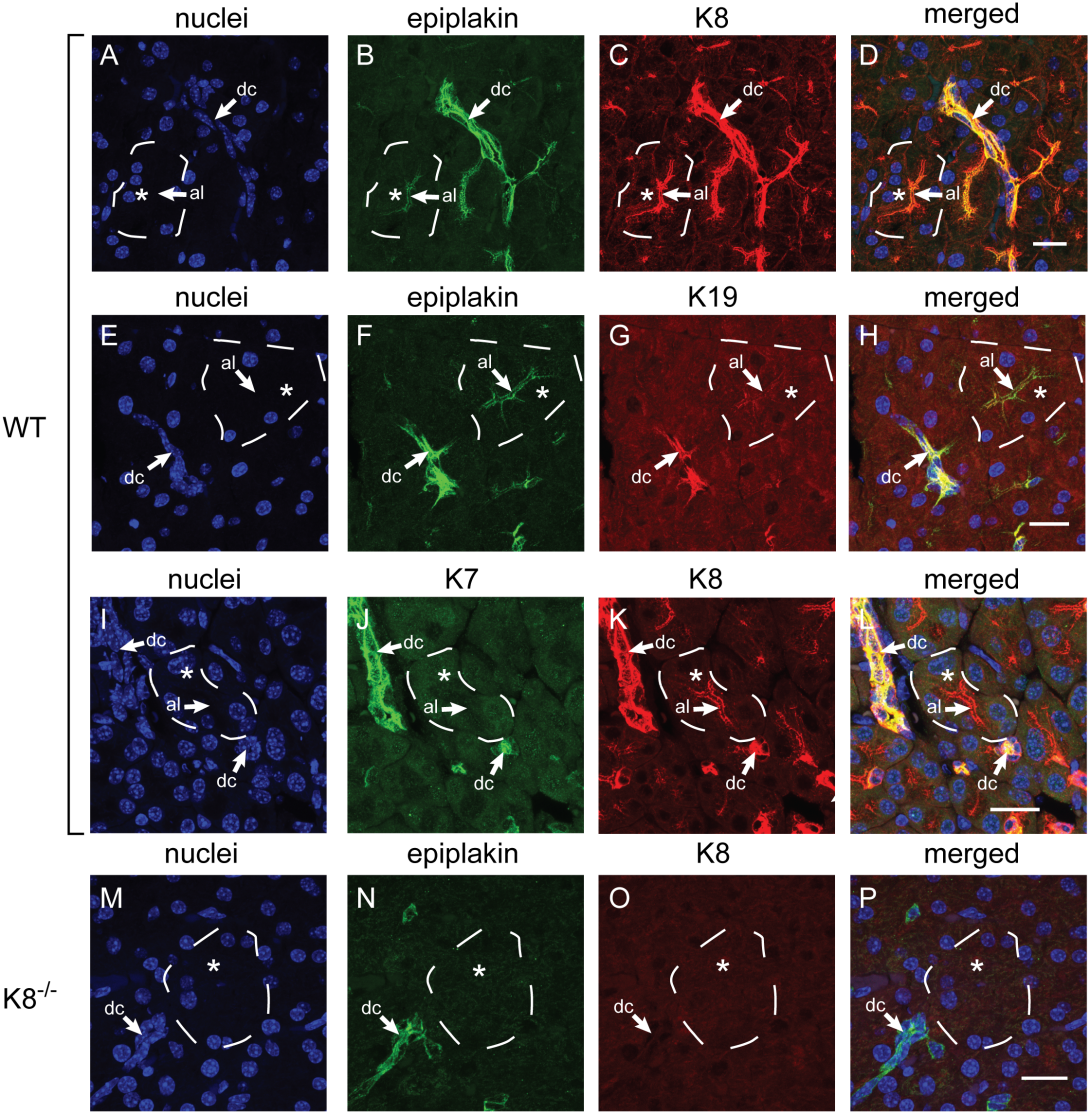
In pancreas, epiplakin colocalizes with ductular and acinar keratins and its apicolateral localization in acinar cells is dependent on keratin filaments. (A–L) Immunofluorescence microscopy for epiplakin, K7, K8 and K19 in pancreata of wild-type mice. Nuclear staining is shown for easier distinction of ductal and acinar cells. Epiplakin is expressed in ductal cells (dc) and in the apicolateral compartment (al) of acinar cells (B, F), where it colocalizes with K8 and K19 (C, G). K7 signals were found in ductal cells but were absent from acinar cells (J). (M–P) Immunofluorescence microscopy for epiplakin and K8 in pancreata of K8-deficient (K8^−/−^) mice. In K8^−/−^ pancreata, epiplakin is expressed in ductal cells but is absent from acinar cells (N). Whole acini are outlined by dashed lines for orientation. Asterisks indicate cytosolic/perinuclear areas. Scale bars, 20 µm.

The keratin-binding protein epiplakin was reported to be robustly expressed in the exocrine pancreas [4], [6]. To investigate epiplakin’s expression pattern and its colocalization with individual keratins in the exocrine pancreas in detail, murine tissue sections were subjected to immunofluorescence microscopy using antibodies to epiplakin and the pancreatic keratins K8, K19, and K7. We found that, besides its strong expression in ductal cells, epiplakin was also expressed in acinar cells where it almost always colocalized with K8-positive apicolateral filaments (Fig. 1B, C, D). Almost no epiplakin was found close to faint K8 filaments which were located in the cytoplasm throughout the acinar cells (Fig. 1B, C, D). Epiplakin’s localization in the exocrine pancreas parallels that of K19, which is restricted to ductal cells and the apicolateral compartment of acinar cells (Fig. 1F, G, H). However, the apicolateral localization of epiplakin in acini was independent of K19 as immunofluorescence microscopy on tissue sections derived from K19-deficient mice showed that lack of K19 alone does not influence epiplakin’s localization in acini (Fig. S1B, F). In wild-type mice, K7 could be detected in ductal cells only and was absent from acinar cells (Fig. 1J). As expected, the filament-associated subcellular localization of epiplakin in acini seems to depend entirely on its binding partners K8/18 or K8/19, as in acinar cells of K8-deficient mice which are completely devoid of keratin filaments, epiplakin’s expression was weak and its localization diffuse (Fig. 1N–P). In ducts of K8-deficient mice epiplakin was colocalizing with K19-containing filaments which are probably formed by K7/19 dimers.

### Epiplakin and K8 become upregulated during pancreatitis

**Figure 2:**
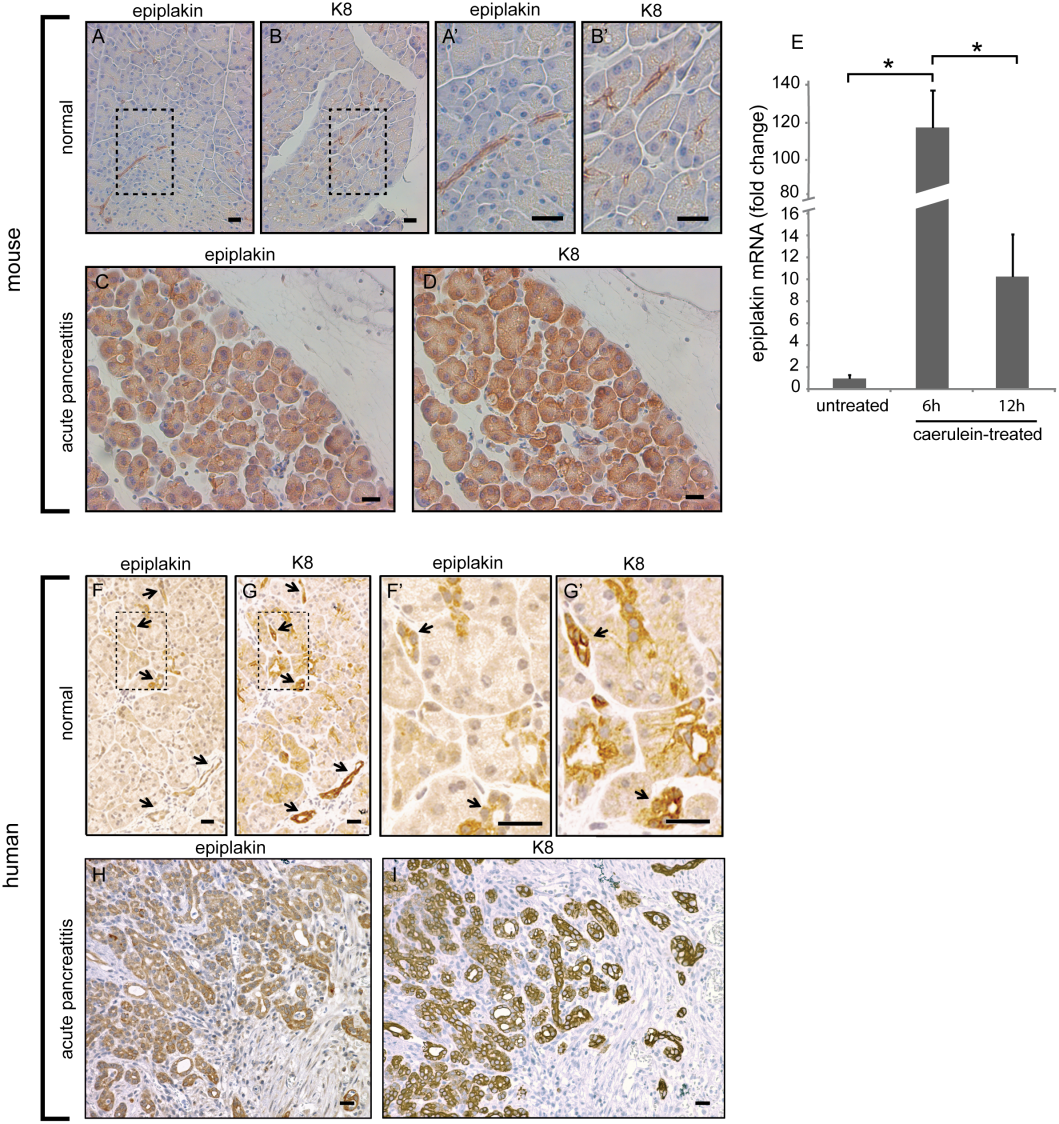
Epiplakin and K8 are upregulated during pancreatitis. Immunohistochemistry for epiplakin and K8 in mouse (A–D) and human (E–H) pancreata. Epiplakin is expressed mainly in ducts of healthy murine pancreas (A and A’). 9 h after pancreatitis induction epiplakin is strongly upregulated which is most apparent in acinar cells (C). Epiplakin closely parallels K8 staining that is predominantly ductal under basal conditions (B and B’) and is robustly upregulated in acinar cells during acute pancreatitis (D). (E) Quantitative RT-PCR analysis of pancreatic RNA extracts confirmed the strong epiplakin overexpression 6 h after pancreatitis induction. Note that epiplakin transcript levels are already significantly decreased at the 12 h timepoint. 3 mice per group and timepoint were used and the values were normalized to the epiplakin levels in untreated mice, which were arbitrarily set as 1. Data are expressed as mean; error bars represent the s.e.m.; n = 3; *, p ≤ 0.05. (F–I) In healthy adult human pancreata, epiplakin is detected primarily in small ducts (arrows in F and F’), where it colocalizes with K8 as seen on consecutive sections (arrows in G and G’). K8 is also expressed in the apical compartment of acinar cells. In acute necrotizing pancreatitis, acinar overexpression of epiplakin (H) and K8 (I) occurs. Rectangles in A, B, F, and G highlight areas that are shown at a higher magnification in A’, B’, F’, and G’, respectively. Scale bars, 25 µm.

K8 expression was shown to be increased during acute pancreatitis before [14]. To investigate whether epiplakin expression was upregulated in a similar manner we analyzed pancreata from mice suffering from caerulein-induced pancreatitis by immunohistochemistry. In untreated control samples prominent epiplakin staining was detected in duct cells whereas its apicolateral localization in acinar cells revealed by immunofluorescence microscopy (Fig. 1B) was hardly detectable using this method (Fig. 2A, A’). Nine hours after the induction of pancreatitis, epiplakin and K8 signals were strongly increased (Fig. 2C, D) compared to basal levels (Fig. 2A, B). Moreover, at this stage of disease strong epiplakin signals were not only detected in ductal cells but also throughout acinar cells (Fig. 2C). Notably, the upregulation was also found in areas without prominent inflammatory infiltrates. Strong epiplakin upregulation six hours after induction of pancreatitis was confirmed on transcriptional level by quantitative real time PCR (Fig. 2E). Interestingly, epiplakin transcript levels were already significantly decreased at the twelve hours timepoint. Immunohistochemistry on normal human pancreatic tissue revealed a similar expression pattern of epiplakin and K8 as in mice (Fig. 2F, F’, G, G’). In samples from patients suffering from acute necrotizing pancreatitis, epiplakin and K8 expression was significantly upregulated and a dramatic increase in epiplakin expression was detected in acinar cells (Fig. 2H, I).

**Figure 3:**
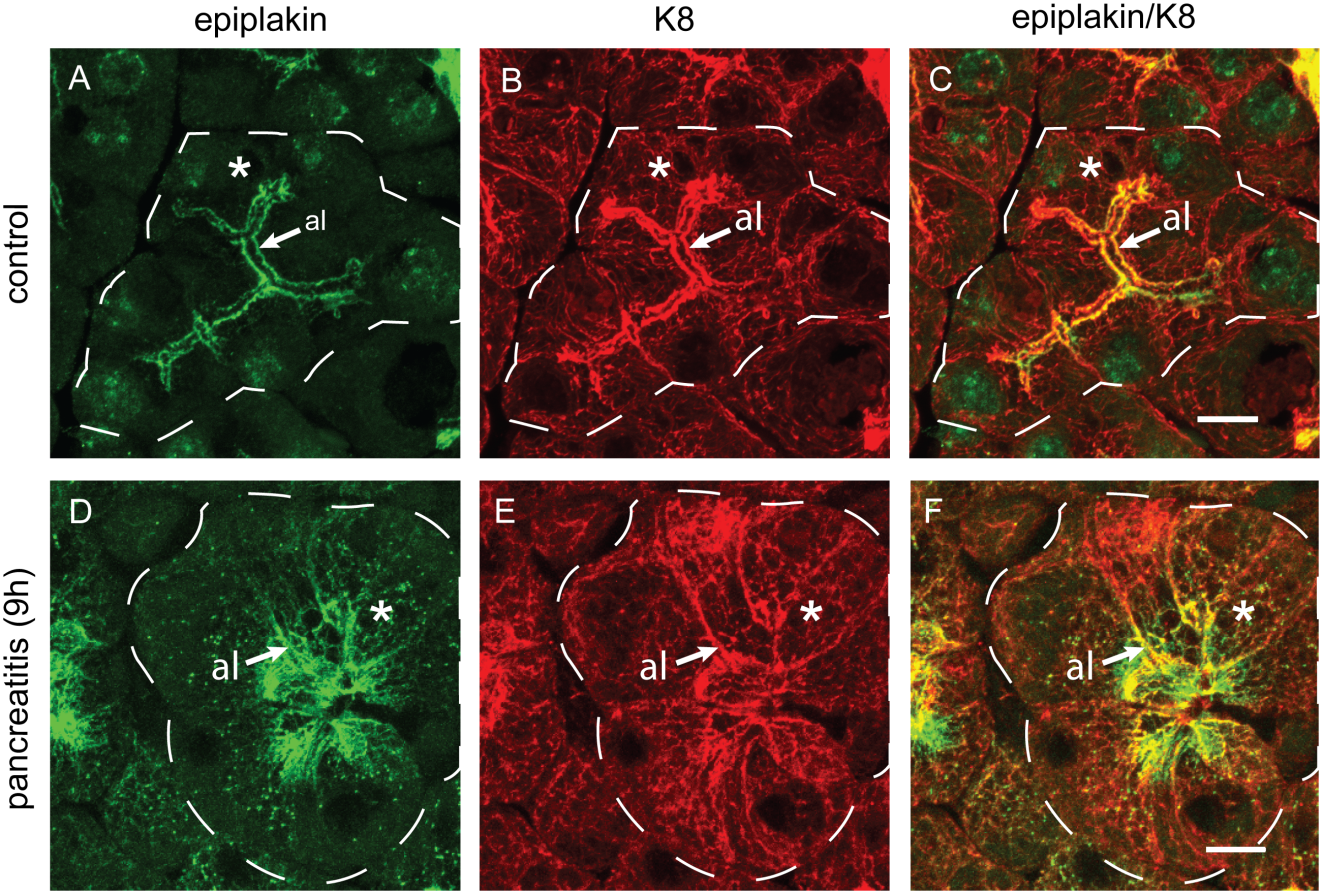
Subcellular localization of epiplakin in acinar cells is altered during pancreatitis-induced keratin reorganization. In healthy pancreas (control) epiplakin is found at the apicolateral compartment (al) of acinar cells (A) colocalizing with apicolateral K8 filaments (B, C). Faint K8 filaments are found in the cytoplasm throughout the acinar cell (B), where epiplakin is hardly detectable (A). 9 h after induction of experimental pancreatitis epiplakin and K8 undergo a similar redistribution (F), but compared to epiplakin (D), K8 is more homogeneously distributed throughout the cytoplasm of acinar cells (E). Whole acini are outlined by dashed lines for orientation. Asterisks indicate cytosolic/perinuclear areas. Scale bars, 10 µm.

For a high resolution microscopic analysis of keratin’s and epiplakin’s localization during pancreatitis, we subjected murine tissue sections from mice to immunofluorescence microscopy using antibodies to epiplakin and K8. Similarly to our findings obtained by immunohistochemistry, upregulation of both epiplakin and K8 was seen nine hours after the induction of pancreatitis (Fig. 3D, E). At this stage of disease, an intense apicolateral localization of epiplakin, but notably also epiplakin-decorated keratin bundles extending further into acinar cells, were observed (Fig. 3D, E, F). In addition, during acute pancreatitis, K8 lost its prevalent apicolateral localization in acinar cells and was found evenly distributed throughout the cell (Fig. 3E). These findings are in line with previous reports describing keratin filament disruption and reorganization immediately after the start of caerulein injections [17].

### Acute pancreatitis is aggravated in EPPK^−/−^ mice

**Figure 4:**
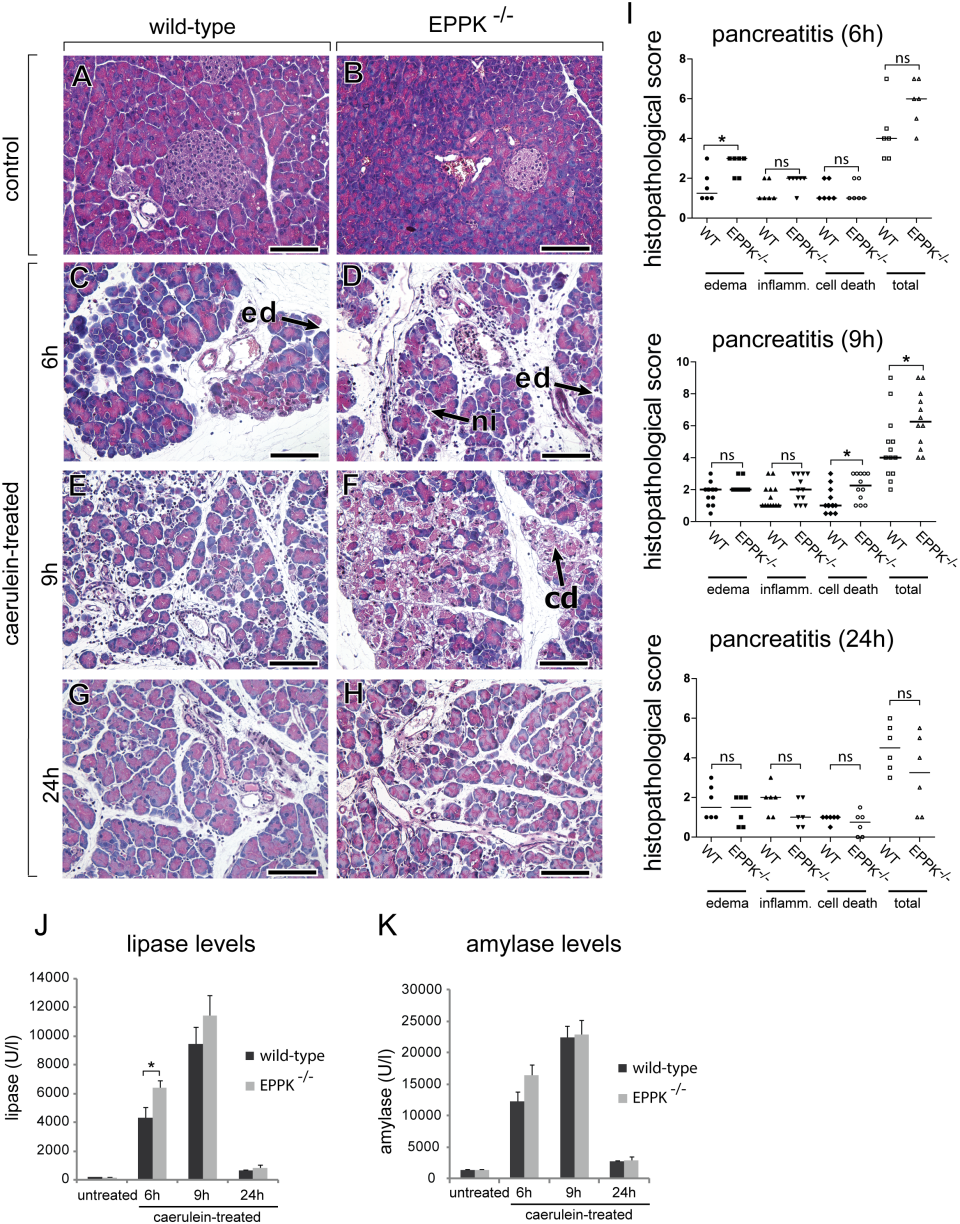
Epiplakin deficiency aggravates caerulein-induced murine pancreatitis. Histological (A–H), morphometrical (I) and serological (J) analyses of caerulein-induced pancreatitis in wild-type and EPPK^−/−^ mice. 6 h after induction of pancreatitis (C, D), EPPK^−/−^ pancreata developed increased edema (ed) and neutrophil infiltration (ni). 9 h after the first caerulein injection (E, F), EPPK^−/−^ pancreata displayed more cell death (cd). Control pancreata showed no obvious abnormalities (A, B) and the caerulein-treated pancreata largely recovered after 24 h in both genotypes (G, H). Scale bars, 100 µm. (I) Histopathological evaluation of wild-type (WT) and EPPK^−/−^ mice pancreatic sections 6 h, 9 h, and 24 h after the induction of pancreatitis. EPPK^−/−^ mice showed increased edema formation 6 h after pancreatitis induction and displayed significantly more cell death and a higher overall pancreatitis score at the 9 h timepoint compared to their wild-type littermates. 24 h after induction no significantly different histopathological scores were found for wild-type and EPPK^−/−^ mice. Horizontal bars represent the median; each symbol represents data from one animal; ns, not significant; n = 12; *, p ≤ 0.05. (J) Serum lipase levels (units per liter) confirm the development of a stronger tissue injury in EPPK^−/−^ mice 6 h after the induction of experimental pancreatitis. (K) Serum amylase levels (units per liter) of EPPK^−/−^ mice during pancreatitis were, although slightly increased, not significantly elevated compared to their wild-type littermates. Data are expressed as mean; error bars represent the s.e.m.; n = 4 for untreated, n = 6 for 6 h and 24 h, and n = 12 for the 9 h timepoint; *, p≤0.05.

Epiplakin’s parallel upregulation with keratin during acute pancreatitis suggested a role of this protein in pancreatic injury. To test this assumption, we analyzed the severity of caerulein-induced acute pancreatitis in EPPK^−/−^ mice and their WT littermates. Deletion of epiplakin in pancreata of EPPK^−/−^ mice was confirmed by immunoblotting and immunohistochemistry (Fig. S2A, B). Histological observations and serum levels from lipase and amylase from unstressed wild-type and EPPK^−/−^ mice manifested no differences (Fig. 4A, B, J). During induced pancreatitis, already six hours after caerulein administration, EPPK^−/−^ pancreata developed increased edema (Fig. 4C, D, I). The highest degrees of damage were observed nine hours after start of caerulein-administration as reflected by histology (Fig. 4E, F, I) and serum lipase levels (Fig. 4J). At this timepoint, pancreata of EPPK^−/−^ mice showed significantly increased areas of necrotic tissue compared to littermate controls (Fig. 4E, F, I). In addition, edema and inflammation both showed a trend towards elevated scores nine hours after induction of pancreatitis in EPPK^−/−^ mice; however, the difference did not reach statistical significance with *p* = 0.08 and *p* = 0.16, respectively (Fig. 4I). The summation of all these parameters into a total score showed a significant aggravation of the disease (*p* = 0.03) in EPPK^−/−^ mice (Fig. 4I). Compared to wild-type littermates, EPPK^−/−^ mice displayed significantly elevated serum lipase levels six hours after induction of pancreatitis. The lipase levels measured at the nine hours timepoint were still showing a tendency towards higher values in EPPK^−/−^ mice (Fig. 4J). Being aware that the differences in lipase levels obtained for wild-type and EPPK^−/−^ mice are minor, we also measured serum amylase levels. Although the differences for wild-type and EPPK^−/−^ mice were not significant, the amylase values at the six hours timepoint showed a clear trend towards higher levels in EPPK^−/−^ mice (Fig. 4K). 24 hours after induction of pancreatitis, serum lipase and amylase levels had returned to almost basal levels, and histopathology showed no significant differences between EPPK^−/−^ mice and their littermates (Fig. 4G, H, I, J, K).

### Aggravated pancreatitis in EPPK^−/−^ mice is not caused by impaired cell-cell junctions, altered keratin expression levels, zymogen granule abnormalities, or impaired amylase release capability

**Figure 5:**
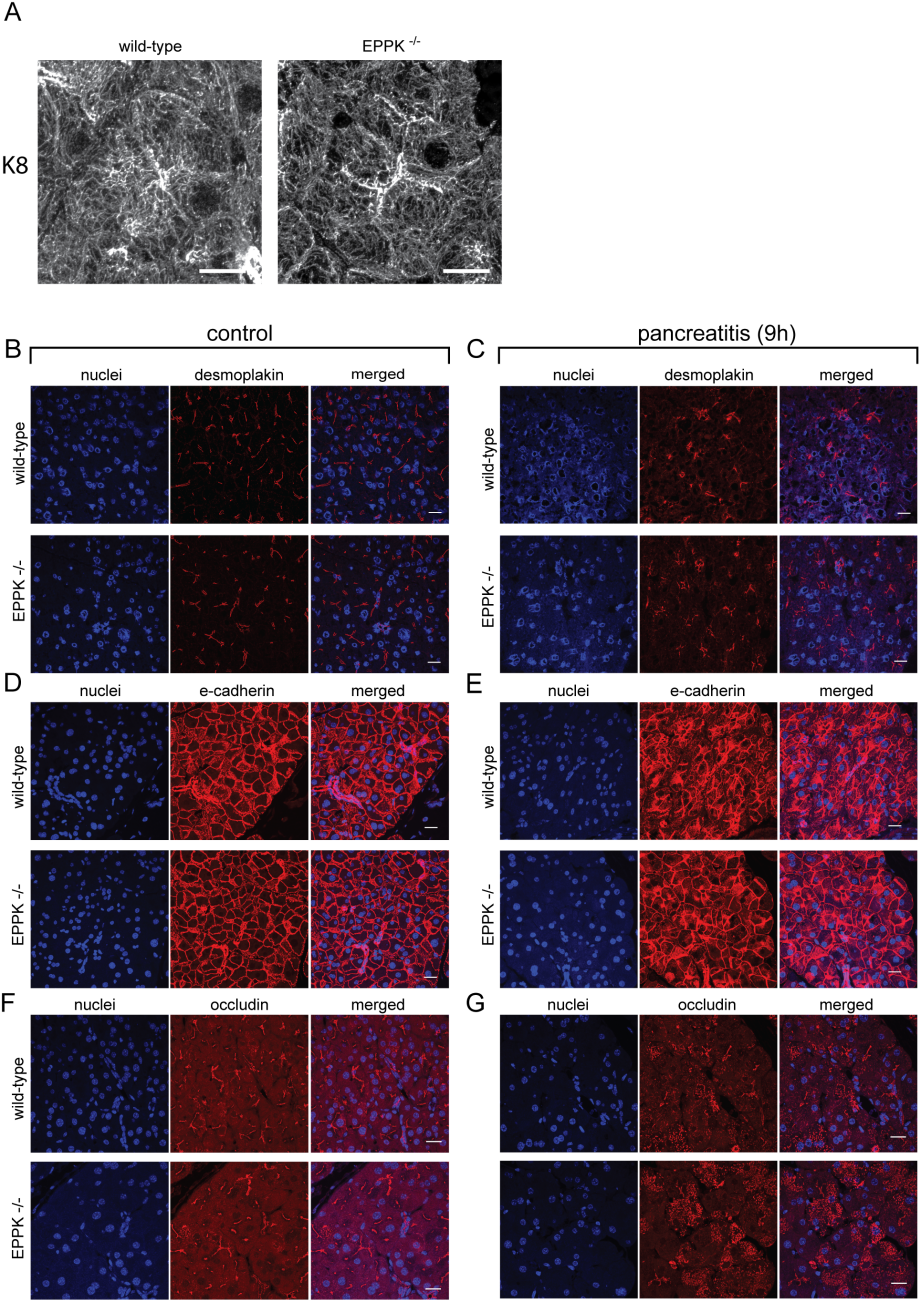
EPPK^−/−^ mice show no abnormalities regarding their acinar keratin organization and junctional complexes. (A) Immunofluorescence microscopy for K8 in pancreata from wild-type and EPPK^−/−^ mice reveal no differences in keratin network structure of acinar cells. (B–E) Immunofluorescence microscopy analysis using antibodies to desmoplakin (B, C) e-cadherin (D, E) and occludin (F, G) shows no differences in intensity and localization of these junctional proteins between wild-type and EPPK^−/−^ mice in unstressed tissue (B, D, F) and during pancreatitis (C, E, G). Note the disassembly of desmoplakin- and occludin-positive structures during pancreatitis in both wild-type and EPPK^−/−^ mice (B–E). Scale bars, 20 µm.

The unique structure of epiplakin comprising multiple keratin-binding PRDs suggests that keratins are epiplakin’s most important and probably sole binding partners. To identify molecular reasons for the pancreatitis phenotype observed in EPPK^−/−^ mice we focused on the investigation of keratin filament organization in acinar cells. It has previously been demonstrated that EPPK^−/−^ mice show no obvious spontaneous phenotypes and that, in stratified epithelia, no abnormalities in the keratin network structure could be detected [10], [11]. Similarly, we did not find any alterations of keratin network architecture in pancreatic acinar cells from unstressed EPPK^−/−^ mice (Fig. 5A). Furthermore, expression and localization of the cell-cell adhesion proteins desmoplakin, e-cadherin, and occludin were found to be unaltered in healthy EPPK^−/−^ mice, indicating unimpaired desmosomes, tight and adherens junctions (Fig. 5B, D, F). Similarly to untreated pancreata no differences in localization and expression of these proteins were found in diseased pancreata of EPPK^−/−^ mice compared to their wild-type littermates, indicating that epiplakin deficiency does not impair these cell-cell junctions during pancreatitis (Fig. 5C, E, G). In addition, no major alterations in the pancreatic protein levels of the plakin protein family members plectin and periplakin as well as the cell-cell adhesion proteins e-cadherin and occludin were detected (data not shown).

Pancreata of transgenic mice overexpressing keratins at high levels display pathological abnormalities [15], [16]. To investigate whether altered keratin levels or modified keratin upregulation during pancreatitis might be responsible for aggravated pancreatitis in EPPK^−/−^ mice we compared pancreatic keratin levels in lysates from both genotypes by immunoblotting. In untreated EPPK^−/−^ and wild-type mice the protein levels of K8, K18, and K19 were similar (Fig. S3A). An equal upregulation of K8, K18, and K19 proteins in wild-type and EPPK^−/−^ lysates was also observed during acute pancreatitis (Fig. S3A, B). Since K7 levels were too low to be detected by immunoblotting, they were analyzed using immunofluorescence microscopy. Nine hours after induction of pancreatitis, comparable ductal expression levels of K7 were seen in both genotypes.

**Figure 6:**
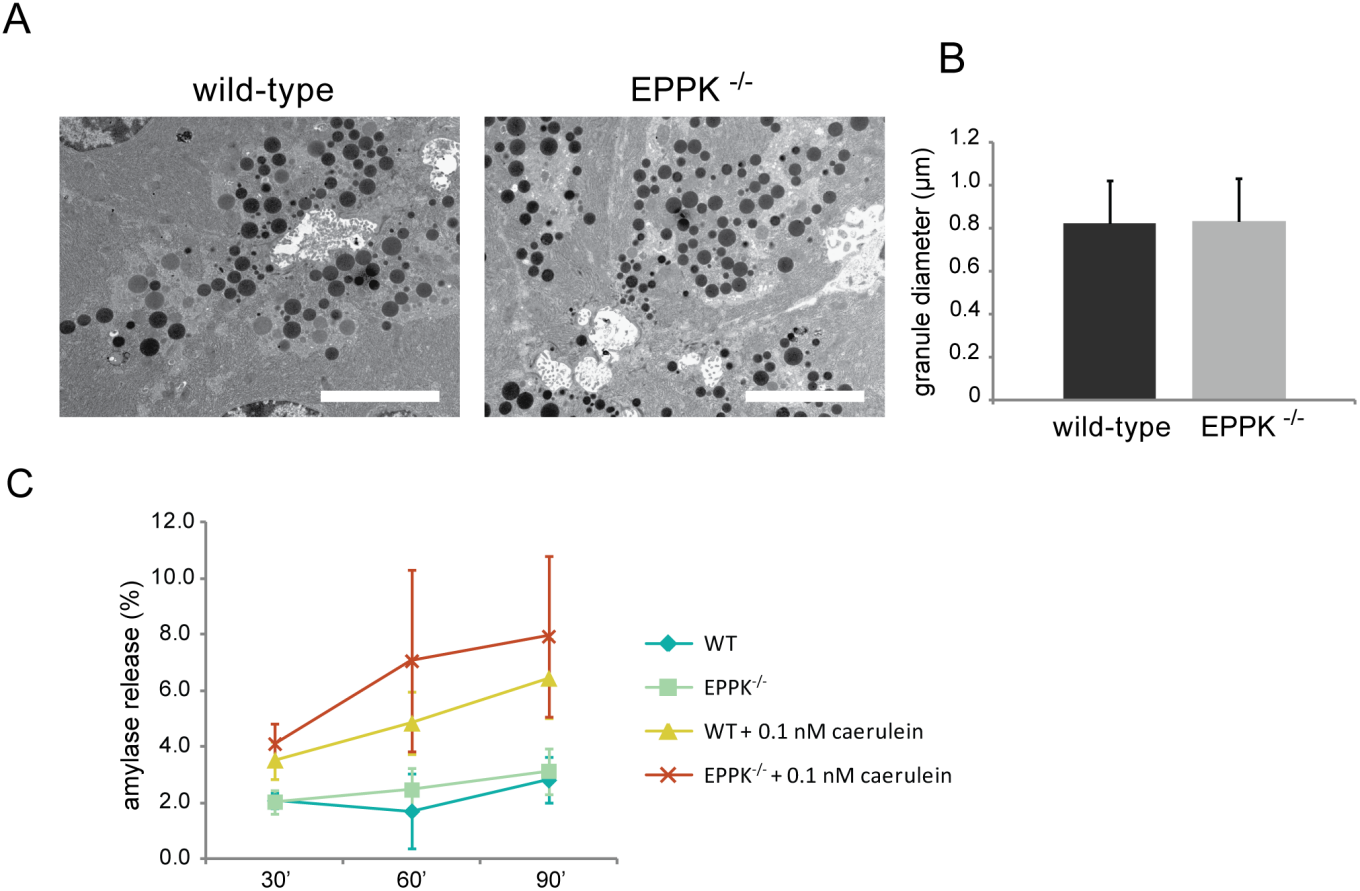
Loss of epiplakin does not impair pancreatic enzyme secretion. (A) Zymogen granules in wild-type and EPPK^−/−^ mouse pancreata were analyzed by transmission electron microscopy. Scale bars, 5 µm. (B) Average diameter of granules was determined by quantification of at least 4 independent sections from 2 animals per genotype. At least 200 zymogen granules were measured per mouse. No significant difference in zymogen granule diameter was found. Data are expressed as mean; error bars represent the SD. (C) Spontaneous and caerulein-induced amylase release of pancreatic lobules isolated from wild-type and EPPK^−/−^ littermates was measured at 3 timepoints after incubation. Values represent amylase release as percentage of total lobular amylase content. Note that no significant differences were found in spontaneous as well as stimulated secretion in wild-type and EPPK^−/−^ mice. Data are expressed as mean; error bars represent the s.e.m.; n = 3 for 60′ and 90′, n = 6 for 30′.

In transgenic mice overexpressing K8/18 and consequently developing pancreatic injury, acinar zymogen granules were found to be smaller, dislocalized and more numerous compared to control animals [15]. To investigate whether altered zymogen granules were responsible for aggravated pancreatitis in EPPK^−/−^ mice, unstressed pancreatic acini from wild-type and EPPK^−/−^ mice were subjected to electron microscopy (Fig. 6A). The evaluation of zymogen granule size showed no differences between WT and EPPK^−/−^ mice (Fig. 6B). To test whether EPPK^−/−^ mice displayed impaired zymogen granule release, spontaneous as well as caerulein-triggered amylase secretion was quantified in pancreatic lobules isolated from EPPK^−/−^ and wild-type mice. Overall, the obtained values for induced amylase release were rather low, however, no significant differences were found for EPPK^−/−^ mice and their wild-type counterparts (Fig. 6C).

### During pancreatitis, EPPK^−/−^ mice show more acini with keratin granules

**Figure 7:**
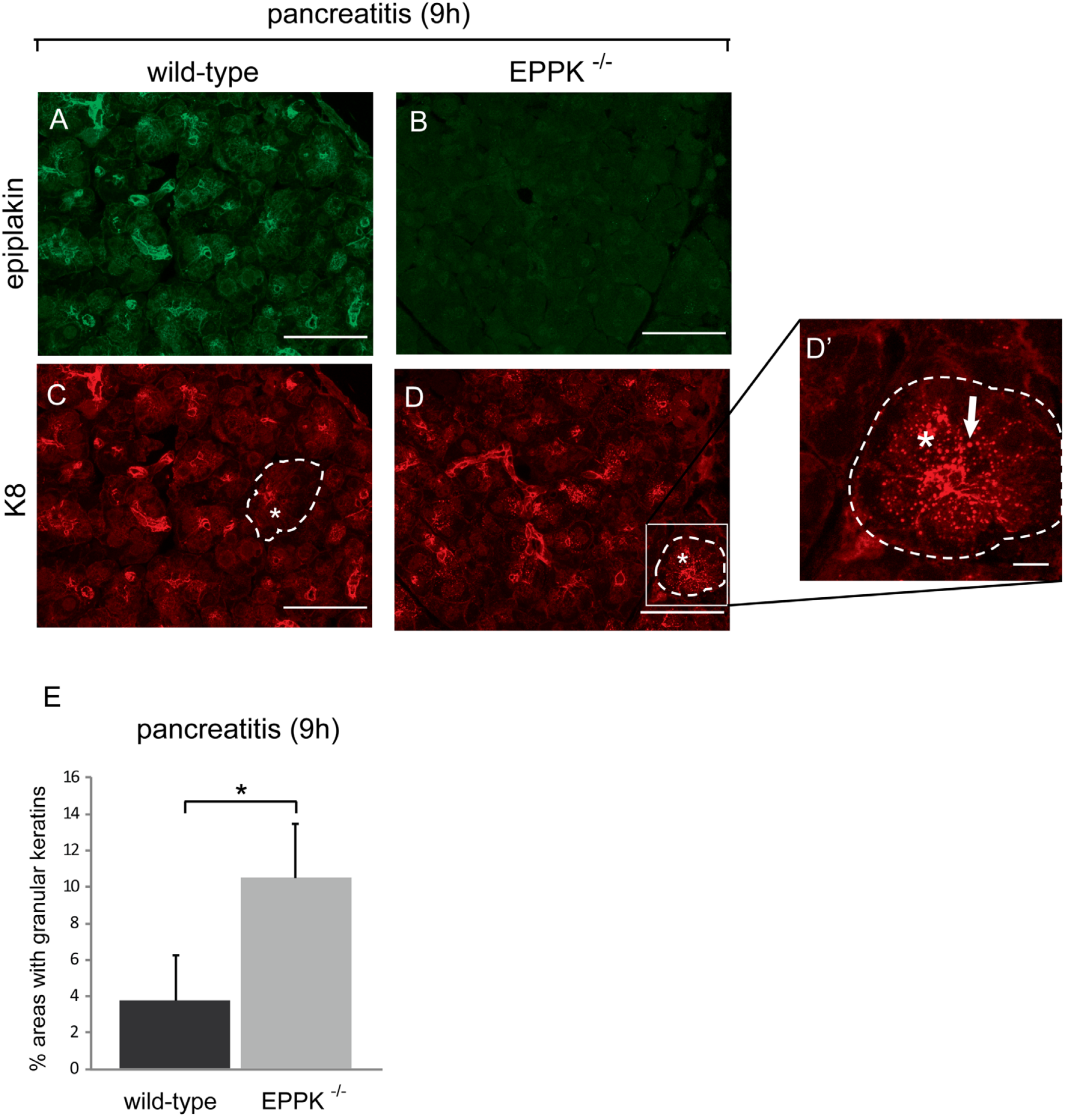
Increased numbers of keratin aggregations in pancreatic acinar cells of EPPK^−/−^ mice during caerulein-induced pancreatitis. (A–D) Immunofluorescence microscopy of epiplakin (A, B) and K8 (C, D) in pancreata of wild-type and EPPK^−/−^ littermates 9 h after the first caerulein injection. Note the increased occurrence of keratin granules in pancreata from EPPK^−/−^ mice (D, D’). Dotted lines indicate individual acini. Asterisks, cytosolic regions of acinar cells. Arrow, keratin granule. Scale bars, 50 µm (A-D); 10 µm (D’). (E) Statistical evaluation of pancreatic areas showing keratin granules during acute pancreatitis. 4.5 mm^2^ of each pancreas section were scored for the occurrence of acinar cells displaying keratin granules. Data are expressed as mean; error bars represent the s.e.m.; n = 6; *, p≤0.05.

During caerulein-induced acute pancreatitis, keratin breakdown and reorganization begin already one hour after the first drug administration [17]. Subsequently, new cytoplasmic keratin filaments begin to form. Phosphorylation of keratins has an important influence on keratin filament reorganization [22]. Thus we analyzed keratin phosphorylation levels in healthy and diseased pancreata which exhibited variations between individual mice but no statistically significant differences between wild-type mice and their EPPK^−/−^ littermates were found (data not shown). As mentioned above, no differences in the organization of keratin filaments were detectable in untreated acinar cells from wild-type and EPPK^−/−^ mice. Three hours after induction of pancreatitis only a few remaining keratin filaments were found in pancreatic acinar cells and even the thick apicolateral bundles were thinner and appeared unstructured (Fig. S4). After six hours acinar cells were still lacking most of the keratin filaments and displayed only thin apicolateral bundles (Fig. S4). In wild-type cells, nine hours after the induction of pancreatitis, acinar keratin filaments had begun to reassemble (Fig. 7C, Fig. S4). However, approximately 4% of wild-type acinar cells were found to contain granular keratin aggregates. Interestingly, acinar cells displaying aggregates were significantly more frequent (∼10%) in EPPK^−/−^ pancreata (Fig. 7D, D’, E). These findings indicate that in EPPK^−/−^ mice pancreatitis-induced keratin reorganization is impaired.

## Discussion

Previously, expression of epiplakin in pancreas was reported to be confined to ducts and the smaller centroacinar duct cells and that during caerulein-induced pancreatitis an expansion of epiplakin-expressing cells can be observed [6]. In this study we show that epiplakin is expressed not only in ductal cells of the pancreas but also in pancreatic acinar cells where it is localized close to the apicolateral membrane. Our data show that during acute pancreatitis epiplakin is upregulated in acinar cells and that its subcellular localization is altered. The distinct apicolateral localization of epiplakin in unstressed acinar cells parallels the main localization of keratins in these cells. This localization does not depend on K19 alone as K19-deficient acinar cells display unaffected epiplakin localization. However, the filament-associated localization of epiplakin in acinar cells seems to completely depend on its binding partner keratin as it shows diffuse subcellular localization in keratin filament-free acini of K8 null mice. This finding also indicates that besides acinar keratins, epiplakin does not bind to other proteins prominently localized at junctional complexes or organelles and does not interact with other cytoskeletal filament systems.

Our data show that, during caerulein-induced acute pancreatitis, epiplakin and keratins are upregulated in a concerted manner and that deficiency of the keratin-binding protein epiplakin aggravates the disease, albeit the increase in severity is modest. In line with findings in stratified epithelia, unstressed pancreatic tissues of EPPK^−/−^ mice show no obvious abnormalities and pancreatitis disease parameters are unchanged compared to wild-type littermates. In previous experiments we could show that epiplakin deficiency in skin does not impair cellular junctions or mechanical properties of cells [10]. Kokado and colleagues reported that epiplakin-deficient corneal epithelium exhibits fragility against mechanical intervention and reduced expression of e-cadherin [23]. In pancreas our data show no differences in localization and expression of desmoplakin, e-cadherin, and occludin in both healthy and diseased pancreata from EPPK^−/−^ mice and their wild-type littermates. These findings indicate that cellular junctions are unaffected in EPPK^−/−^ mice even during pancreatitis.

Excessive overexpression of keratins is well tolerated in liver but not in pancreas [15]. For example, transgenic mice significantly overexpressing human K8 developed progressive chronic pancreatitis and showed increased apoptosis [16]. We could rule out the possibility that the lack of epiplakin leads to gross differences in expression levels of K7, K8, K18, and K19 which theoretically could have caused the phenotype observed. Another mouse line overexpressing K8 and K18 displayed age-related enhanced vacuolization and atrophy in the exocrine pancreas, accompanied by altered zymogen granule size, number, and localization [15]. However, we could not detect any abnormalities regarding zymogen morphology or cellular distribution in EPPK^−/−^ mice. In addition, EPPK^−/−^ pancreatic lobules showed no constraints regarding their capability to induce caerulein-triggered amylase release in *ex vivo* assays. A mouse line overexpressing mutant human K18 (Arg89→Cys; K18C) shows disrupted cytoplasmic but intact apicolateral keratin filaments in acinar cells [17]. Compared to wild-type littermates, K18C mice exhibited increased basal serum amylase levels but their impaired acinar cytoplasmic keratin filaments did not render these mice more susceptible to caerulein-induced pancreatitis. A possible explanation for this finding was that, as part of the recovery process after caerulein-induced damage, K18C mice, which normally have disrupted cytoplasmic filaments, acquired the ability to form intact filaments most likely by upregulation of their endogenous murine K18 [17]. In contrast to this mutant mouse line, healthy EPPK^−/−^ mice show unaltered acinar keratin filament organization. However, after caerulein-induced pancreatitis accompanied by keratin upregulation and reorganization we observed an aggravated course of disease and more acinar cells displaying keratin granules in EPPK^−/−^ mice. Our interpretation of this finding is that epiplakin is involved in keratin reorganization after stress-induced keratin upregulation.

This theory is supported by the following observations: i) The filament-associated localization of epiplakin in acinar cells is completely dependent on its binding partner keratin as shown in acinar cells devoid of keratin filaments. This finding and epiplakin’s unique structure comprising 16 similar PRDs, most of which bind to keratins [12] suggest that keratins are epiplakin’s most important and probably sole binding partners in acinar cells. ii) During acute pancreatitis epiplakin and keratins are upregulated in a concerted manner in both mice and humans, indicating a close functional relationship. iii) The observed keratin reorganization phenotype showing increased granule formation is detected at the timepoint of maximal keratin reorganization after caerulein-induced pancreatitis. iv) Localization of cell junction-associated proteins appears unchanged in normal and diseased pancreata of EPPK^−/−^ mice, indicating that lack of epiplakin does not impair cell-cell junctions, which could cause the observed pancreatitis phenotype. v) Mice devoid of acinar keratins were reported to show no pancreatic abnormalities compared to their wild-type littermates, even when challenged with caerulein [14]. Given that acinar keratins are dispensable during acute pancreatitis, we doubt that the observed phenotype in EPPK^−/−^ mice is caused by loss of normal keratin functions but rather by gain of functions due to unorganized keratin network. In line with this model are findings from Watson and colleagues [24] who reported that the developmental phenotype of transgenic mice lacking the keratin chaperon Mrj is not due to a deficiency of the normal keratin cytoskeleton but is caused by cytotoxic keratin aggregates that disrupt chorion trophoblast cell organization and function.

Additional experiments are needed to further elucidate the molecular mechanisms by which epiplakin deficiency favors the formation of keratin aggregates. It will be important to investigate whether these aggregates are causing cell death which might lead to the observed aggravated pancreatitis in EPPK^−/−^ mice. In addition, analysis of double knockout mice with epiplakin and keratin deficiency could prove a causal relationship between the formation of keratin granules and severity of caerulein-induced pancreatitis.

## Supporting Information

Figure S1 **Epiplakin’s apicolateral localization does not depend on K19.** (A–H) Formaldehyde-fixed and paraffin-embedded pancreatic sections from K19^−/−^ mice were subjected to immunofluorescence microscopy using antibodies to epiplakin, K8 and K19. Nuclear staining is shown for easier distinction of ductal and acinar cells. Epiplakin staining is found in ductal cells (dc) and apicolaterally (al) in acinar cells (B,F), colocalizing with K8 (D). K19 is absent from ductal and acinar cells (G). Whole acini are outlined by dashed lines for orientation. Asterisks indicate cytosolic/perinuclear areas. Scale bars, 20 µm.
(TIF)

Figure S2 **Characterization of EPPK^−/−^ pancreata.** (A) Immunoblotting of epiplakin from cell lysates of wild-type (WT) and EPPK^−/−^ mouse pancreata. Ponceau staining is shown as loading control. Note that due to the large molecular mass of epiplakin (∼750 kDa), no marker bands were present in the selected areas of the immunoblot. (B) Immunohistochemistry of paraffin sections showing predominant ductal localization of epiplakin in wild-type and lack of epiplakin in EPPK^−/−^ pancreata. Scale bars, 50 µm.
(TIF)

Figure S3 **Loss of epiplakin does not alter pancreatic keratin expression levels.** (A) Immunoblotting visualizes protein levels of K8, K18, and K19 in pancreata of EPPK^−/−^ and wild-type (WT) mice before (control) and 9 h after induction of acute pancreatitis. Ponceau staining shows equal loading. (B) Densitometric quantification of pancreatic keratin levels of EPPK^−/−^ mice 9 h after the induction of pancreatitis relative to that in wild-type mice (100%, dashed line). Note that keratin levels are comparable between wild-type and EPPK^−/−^ samples. Data are expressed as mean; error bars represent the s.e.m.; n≥6.
(TIF)

Figure S4 **Reorganization of epiplakin and the keratin 8 network during experimental pancreatitis.** Formaldehyde-fixed and paraffin-embedded pancreatic sections from wild-type (WT) and EPPK^−/−^ mice were subjected to immunofluorescence microscopy using antibodies to epiplakin and K8. In all images, an individual acinus is outlined by a dotted line. Note the dramatic loss of apicolateral keratin and epiplakin signals in acinar cells 3–6 h after induction of pancreatitis. In wild-type cells, 9 h after the first caerulein injection, a pronounced formation of K8 filaments accompanied by positive epiplakin signals is seen throughout the acinar cell cytoplasm. At this timepoint EPPK^−/−^ acini frequently display keratin aggregations. Arrows and asterisks depict apicolateral keratin bundles and central regions of acinar cells, respectively. Scale bars, 20 µm.
(TIF)
