# Supplementary figures and images for: Epiplakin Deficiency Aggravates Murine Caerulein-Induced Acute Pancreatitis and Favors the Formation of Acinar Keratin Granules
Source: PLoS One. 2014 Sep 18;9(9):e108323. doi: 10.1371/journal.pone.0108323 (PMC4169488; doi:10.1371/journal.pone.0108323)

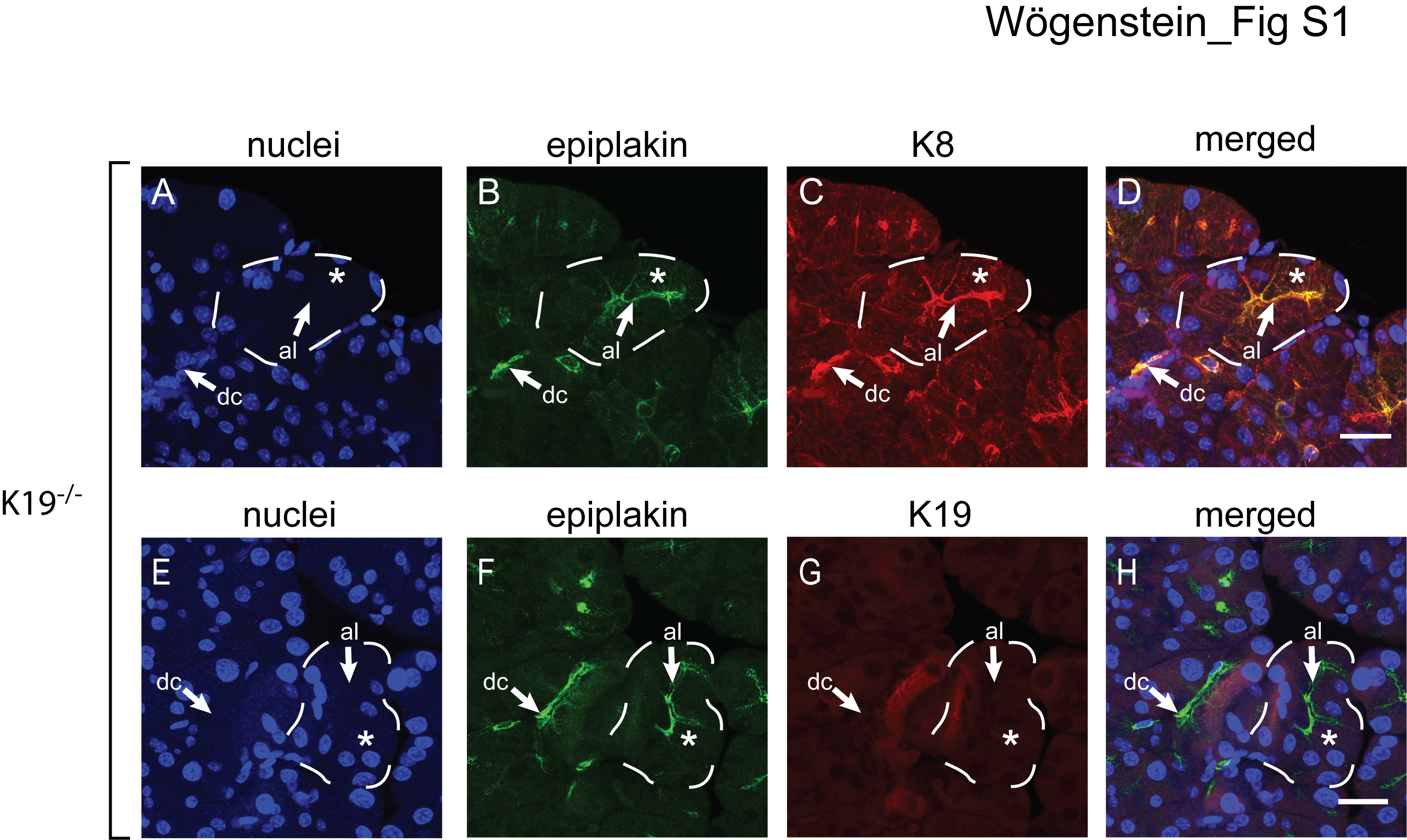

Supplement: Figure S1 — Epiplakin’s apicolateral localization does not depend on K19. (A–H) Formaldehyde-fixed and paraffin-embedded pancreatic sections from K19−/− mice were subjected to immunofluorescence microscopy using antibodies to epiplakin, K8 and K19. Nuclear staining is shown for easier distinction of ductal and acinar cells. Epiplakin staining is found in ductal cells (dc) and apicolaterally (al) in acinar cells (B,F), colocalizing with K8 (D). K19 is absent from ductal and acinar cells (G). Whole acini are outlined by dashed lines for orientation. Asterisks indicate cytosolic/perinuclear areas. Scale bars, 20 µm. (TIF) [file pone.0108323.s001.tif]

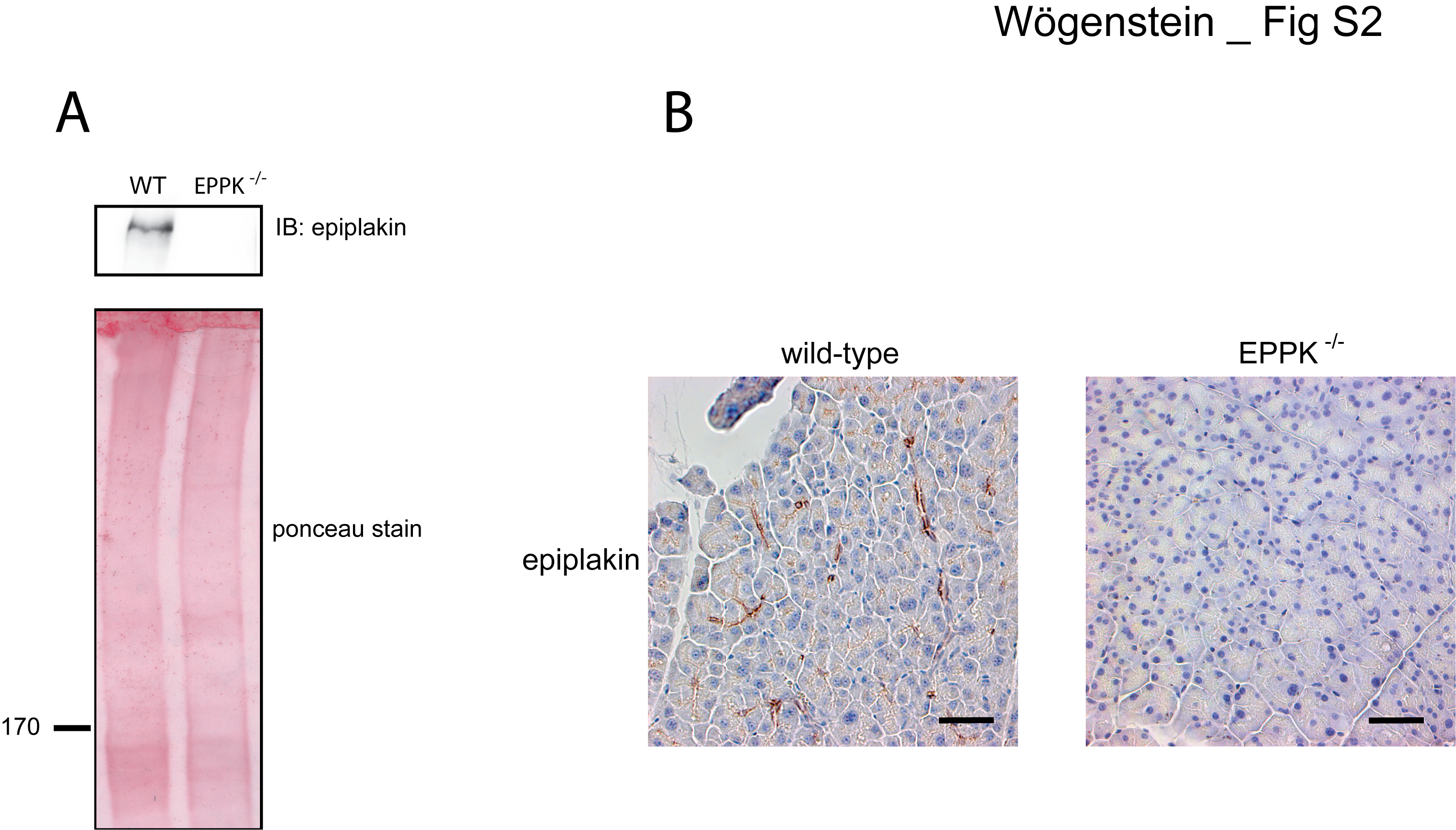

Supplement: Figure S2 — Characterization of EPPK−/− pancreata. (A) Immunoblotting of epiplakin from cell lysates of wild-type (WT) and EPPK−/− mouse pancreata. Ponceau staining is shown as loading control. Note that due to the large molecular mass of epiplakin (∼750 kDa), no marker bands were present in the selected areas of the immunoblot. (B) Immunohistochemistry of paraffin sections showing predominant ductal localization of epiplakin in wild-type and lack of epiplakin in EPPK−/− pancreata. Scale bars, 50 µm. (TIF) [file pone.0108323.s002.tif]

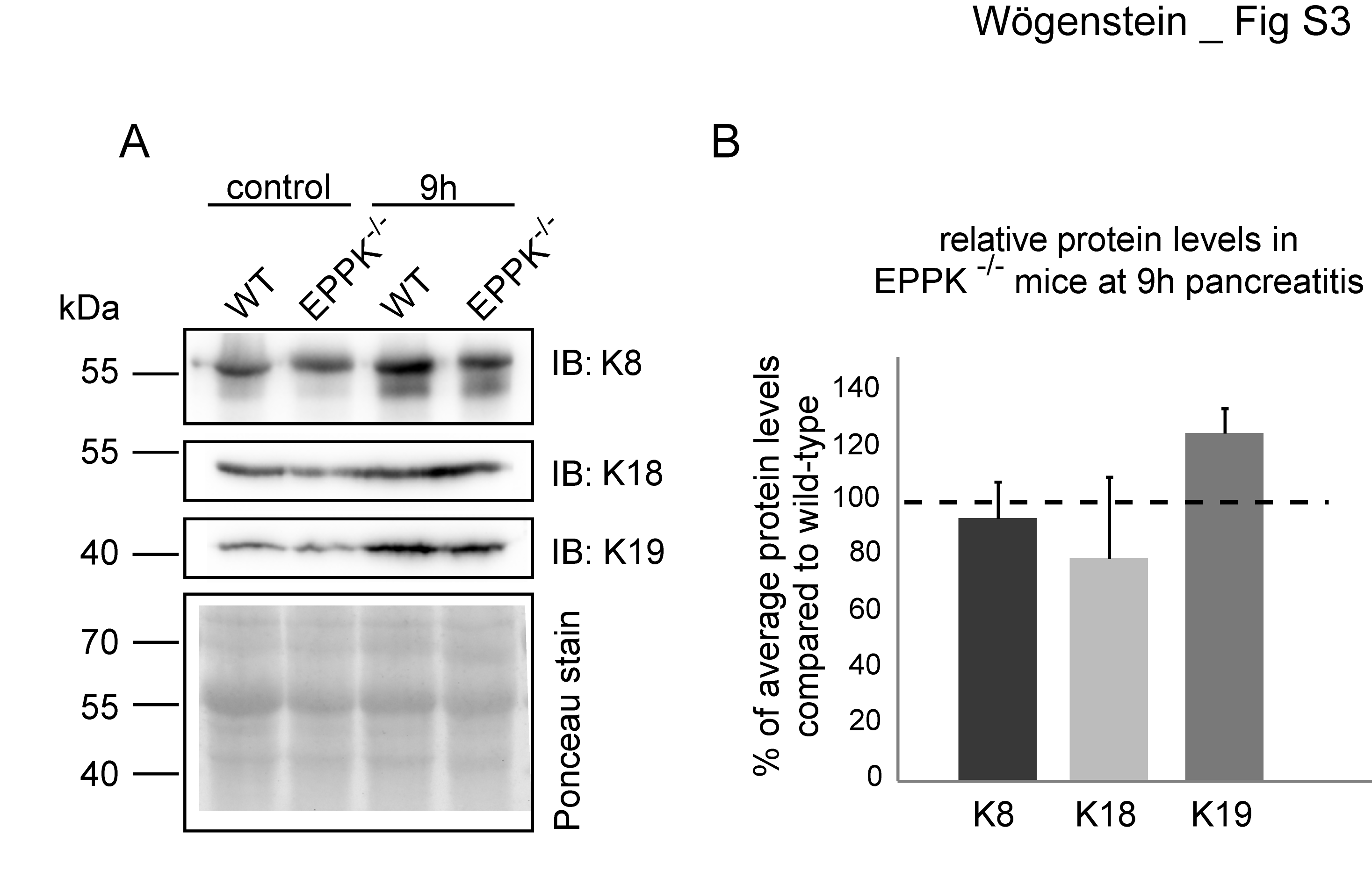

Supplement: Figure S3 — Loss of epiplakin does not alter pancreatic keratin expression levels. (A) Immunoblotting visualizes protein levels of K8, K18, and K19 in pancreata of EPPK−/− and wild-type (WT) mice before (control) and 9 h after induction of acute pancreatitis. Ponceau staining shows equal loading. (B) Densitometric quantification of pancreatic keratin levels of EPPK−/− mice 9 h after the induction of pancreatitis relative to that in wild-type mice (100%, dashed line). Note that keratin levels are comparable between wild-type and EPPK−/− samples. Data are expressed as mean; error bars represent the s.e.m.; n≥6. (TIF) [file pone.0108323.s003.tif]

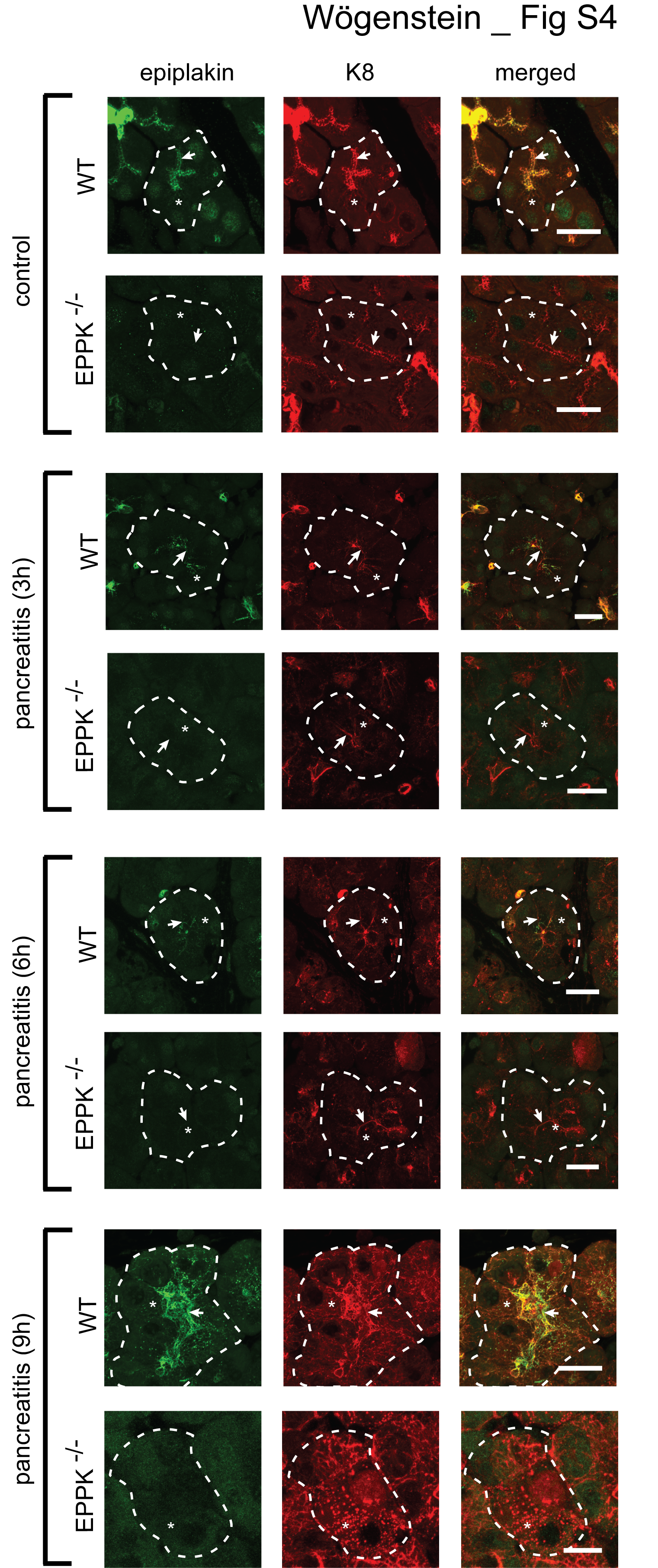

Supplement: Figure S4 — Reorganization of epiplakin and the keratin 8 network during experimental pancreatitis. Formaldehyde-fixed and paraffin-embedded pancreatic sections from wild-type (WT) and EPPK−/− mice were subjected to immunofluorescence microscopy using antibodies to epiplakin and K8. In all images, an individual acinus is outlined by a dotted line. Note the dramatic loss of apicolateral keratin and epiplakin signals in acinar cells 3–6 h after induction of pancreatitis. In wild-type cells, 9 h after the first caerulein injection, a pronounced formation of K8 filaments accompanied by positive epiplakin signals is seen throughout the acinar cell cytoplasm. At this timepoint EPPK−/− acini frequently display keratin aggregations. Arrows and asterisks depict apicolateral keratin bundles and central regions of acinar cells, respectively. Scale bars, 20 µm. (TIF) [file pone.0108323.s004.tif]
